# Differential immunologic response to GnRHa alone vs. dual trigger for final follicular maturation: a proof-of-concept pilot study

**DOI:** 10.3389/frph.2026.1890241

**Published:** 2026-07-10

**Authors:** Raoul Orvieto, Helly Vernitsky, Marina Safanaiev, Dolly Yuster, Dan Dominissini

**Affiliations:** 1 Infertility and IVF Unit, Department of Obstetrics and Gynecology, Chaim Sheba Medical Center, Gray Faculty of Medical and Health Science, Tel Aviv University, Tel-Aviv, Israel; 2 The Tarnesby-Tarnowski Chair for Family Planning and Fertility Regulation, at the Gray Faculty of Medical and Health Science, Tel-Aviv University, Tel Aviv, Israel; 3 Hematology Laboratory, Sheba Medical Center, Ramat-Gan, Israel; 4 Cancer Research Center and Wohl Institute for Translational Medicine, Ramat Gan, Israel

**Keywords:** dual trigger, flow cytometry, GnRHa, hCG, immunologic response, OHSS

## Abstract

**Objective:**

To explore the impact of GnRH agonist (GnRHa) vs. dual trigger (GnRHa plus hCG) for final follicular maturation on peripheral immune profiles in women undergoing oocyte cryopreservation.

**Design:**

Prospective proof-of-concept hypothesis-generating pilot study.

**Setting:**

University-affiliated single center.

**Patients:**

Five healthy women undergoing fertility preservation (*n* = 3 GnRHa trigger, *n* = 2 dual trigger).

**Intervention(s):**

Ovarian stimulation with either GnRHa alone or dual trigger (GnRHa + hCG). Immune profiling was performed at two time points: (1) before trigger, and (2) on the day of oocyte retrieval.

**Main outcome measure(s):**

Percent change (%Δ) in peripheral immune blood T, B, and NK cell populations using flow cytometry.

**Result(s):**

Baseline characteristics were similar. Compared with GnRHa alone, dual trigger was associated with a trend toward lower CD4/CD8 (helper/cytotoxic) T cell ratio, and an apparent increase in highly differentiated cytotoxic (CD57+) cell populations. However, given the small sample size, findings should not be interpreted as definitive but as exploratory trends. There were no cases of OHSS.

**Conclusion(s):**

In this small, exploratory hypothesis-generating study, the addition of hCG to GnRHa trigger was associated with a shift toward a more cytotoxic lymphocyte profile post trigger. Findings require validation in larger, randomized cohorts to better understand their implications for reproductive outcomes and OHSS risk.

## Introduction

Human chorionic gonadotropin (hCG) and gonadotropin-releasing hormone agonist (GnRHa) are widely used triggers for final oocyte maturation in assisted reproductive technology (ART) cycles. hCG acts as a surrogate for the endogenous luteinizing hormone (LH) surge, while GnRHa induces an endogenous surge of both LH and follicle-stimulating hormone (FSH), more closely mimicking the natural cycle (1).

Despite sharing the same clinical endpoint, induction of oocyte maturation, these two approaches differ in their biological effects. hCG's prolonged half-life leads to sustained luteotropic activity, linked to inflammatory mediator upregulation (as vascular endothelial growth factor and other inflammatory cytokines) and elevated risk of ovarian hyperstimulation syndrome (OHSS) (2). Conversely, GnRHa triggers a shorter, more physiological LH/FSH release, resulting in negligible OHSS risk (3, 4). However, in fresh embryo transfer cycles, GnRHa triggers demonstrate lower pregnancy rates and higher early pregnancy loss compared to hCG, possibly owing to luteal phase defects (5). Although an intense luteal support with estradiol and progesterone has been suggested to mitigate GnRHa's adverse effect on implantation (6), this doesn't fully explain the lower live birth rates observed compared to egg-recipients cycles lacking a functional corpus luteum or other clinical observations (7).

There is growing evidence that immune modulation plays a crucial role in follicular development, implantation, and early pregnancy (8, 9). Differences in immune activation in response to different maturation triggers may underpin, at least partly, observed disparities in ART outcomes. For example, systemic inflammation, as measured by markers such as C-reactive protein (CRP) and interleukins, appears lower after GnRHa than hCG trigger (2, 4); however, little is known about their effects on discrete immune cell populations, particularly in the context of oocyte cryopreservation where the confounder of pregnancy or embryo transfer is absent.

Given the immunologic complexity of ART cycles, this exploratory *proof-of-concept pilot study* sought to compare peripheral lymphocyte population shifts in women undergoing GnRHa vs. dual trigger (GnRHa plus hCG) for oocyte cryopreservation. Our aim was to generate testable hypotheses regarding immune responses and their potential reproductive implications.

## Patients and methods

### Study population

This prospective pilot study included five healthy women planning oocyte cryopreservation at our academic infertility unit. Patients with chronic illness or receiving chronic medical treatment were excluded. Each patient was thoroughly counseled and gave informed consent before participating in the study. The Institutional Review Board (SMC-2536-15) approved the protocol.

### Ovarian stimulation(OS) and trigger allocation

All women underwent OS using the multiple dose GnRH-antagonist protocol, as previously described (10). Briefly, following the confirmation of quiescent ovaries and low serum estradiol (E2) levels on days 2/3 of menstruation, FSH was administered subcutaneously at doses ranging from 225 to 300 IU, tailored to age and previous responsiveness. The gonadotropin dosage was adjusted individually according to serum E2 levels and vaginal ultrasound measurements of follicular diameter, obtained every 1 or 2 days. Cetrorelix (Cetrotide; Serono Laboratories), 0.25 mg daily subcutaneously, was added when the leading follicle reached 13–14 mm diameter, and continued until the day of final follicular maturation trigger. When at least three mature > 17 mm follicles were obtained, patients were assigned to receive an injection of either 0.2 mg triptorelin (Decapeptyl; Ferring, Malmo, Sweden) (GnRH group), or 250 mg recombinant hCG (Ovitrelle; Serono Laboratories) combined with 0.2 mg triptorelin (dual trigger). Trigger allocation was based on physician discretion following individual ovarian response (11), introducing potential selection bias that is an acknowledged study limitation. Transvaginal oocyte retrieval was performed 36 h post-trigger.

Transvaginal oocyte retrieval was performed 36 h. post trigger under ultrasound guidance. The study required no modification of our routine IVF protocols.

For the purposes of the study, in addition to routine monitoring during the OS cycle, blood samples were drawn to determine patients' immune profile at two time points: (1) the day of or prior to final follicular maturation trigger (Day-trigger); and (2) the morning before ovum pick-up (Day-OPU). Trigger allocation was determined by treating physicians based on ovarian response (no randomization).

### Rationale and selection of immune markers

Peripheral blood flow cytometry focused on T lymphocyte subsets (CD3+, CD4+, CD8+), B cells (CD19+), NK cells (CD16+, CD56+), and expression of CD57 on T and NK cells. These populations were selected based on their established or hypothesized roles in reproductive immunology: CD4/CD8 ratio as a reflection of adaptive immunity balance; CD57 expression identifying highly differentiated cytotoxic effectors implicated in implantation, inflammation, and maternal immune adaptation (12, 13). The study did not assess tissue-resident or endometrial immune cells.

### Sample handling and flow cytometry

Peripheral whole blood samples were collected into EDTA tubes and processed within 2 h. A multicolor antibody panel was used to identify T, B, and NK cell populations. Samples were incubated for 15 min at room temperature in the dark with fluorochrome-conjugated monoclonal antibodies: CD57 FITC, CD56 PE, CD16 PE, CD8 PC5.5, CD4 APC a700, CD19 APC a 123 750, CD3 PC7, CD2 PB, CD45 KO. All antibodies were purchased from Beckman Coulter and used according to the manufacturer's recommended concentrations. After incubation, red blood cells were lysed by adding ammonium chloride lysing solution (BD Pharm Lyse) and incubating for 10 min at room temperature. Data acquisition was performed immediately, using the BD FACSLyric Flow Cytometer. Instrument setup and performance tracking were conducted according to the manufacturer's guidelines. Samples were run blinded to the treatment group. Up to 5,000 lymphocyte events were acquired per sample. This acquisition threshold is routinely applied in our laboratory for lymphocyte subset analysis and has demonstrated acceptable reproducibility and accuracy as verified by ongoing internal quality control procedures and participation in the UK NEQAS external quality assessment.

Flow cytometry data were analyzed using BD FACSuite software. The lymphocyte population was identified on the CD45/SSC plot, and a minimum of 5,000 events were acquired. Lymphocyte subsets were analyzed using CD3/CD56 + 16, CD3/CD4, CD3/CD8 dot plots. The various immune cell subsets were identified and expressed as percentages of the total lymphocyte population.

Internal quality control was performed weekly using R&D status flow immunology control. In addition, the laboratory participates in the UK NEQAS immunology external quality assessment program.

### Statistical analysis

Given the limited sample size, non-parametric statistical methods (Mann–Whitney *U*) were applied to analyze percent changes (%Δ) in immune cell populations and hormonal levels pre- and post-trigger. Results are presented as exploratory trends.

## Results

Five women were included: 3 in the GnRHa group, 2 in the dual trigger group. Baseline characteristics, including age, BMI, Day 3 FSH, total gonadotropin dose, stimulation duration, and retrieved oocyte number are summarized in Table 1.

**Table 1:** Patients’ baseline characteristics and ovarian stimulation variables.

Patients	Age (years)	BMI (Kg/m^2^)	Day 3 FSH (IU/L)	Duration of OS	Total FSH dose	# of oocyte retrieved	Day of trigger			Day of OPU		
							E2 (pmol/L)	Progesterone (nmol/L)	LH (IU/L)	E2 (pmol/L)	Progesterone (nmol/L)	LH (IU/L)
GnRH												
1	28	25.6	5.6	10	2,175	25	24,545	2.8	3	9,212	23.2	3.5
2	35	26.3	7	10	2,850	28	12,934	2.6	4.6	6,292	36.8	7.4
3	35	19.6	1	13	4,200	8	8,062	2.6	6.9	3,378	13.8	21.7
Dual trigger												
1	33	26.2	7.4	14	4,650	19	4,202	9.9	1	2,612	60.2	3.7
2	34	25.4	11.4	13	5,850	20	2,766	2.1	7	964	11.4	6.6

Baseline immune profile values varied among individuals; therefore, results at both time points and percent change from pre to post trigger (%Δ between Day-OPU and baseline values: Day-OPU minus Day-trigger divided by Day-trigger*100) are shown in Table 2.‏

**Table 2:** The absolute and percentage difference (%Δ) between the value on Day-OPU (post trigger) and the values on Day-trigger (Day-OPU minus Day-trigger divided by Day-trigger*100).

		Immune profile																					
Patients		WBC	LYMPH%	LYMPH	CD3%	CD3	CD4%	CD4	CD8%	CD8	CD2(T-ER)	CD4/CD8	CD20%	CD20	CD19%	CD19	CD3(CD56 CD16)%	CD25%	CD3CD57%	CD(56 16)%	CD2CD57%	CD57%	FOX P3
GnRH group																							
1	Day trigger	5,780	21.7	1,254	75	94.1	49	615	27	339	85	1.81	9	113	9	113	6	3	12	18	13	14	20.5
1	Day OPU	5,910	19.4	1,147	77	883	52	596	26	298	84	2	9	103	10	115	5	2	8	14	11	11	27
1	%Δ	2.249135	−10.5991	−8.5327	2.666667	838.3634	6.122449	−3.08943	−3.7037	−12.0944	−1.17647	10.49724	0	−8.84956	11.11111	1.769912	−16.6667	−33.3333	−33.3333	−22.2222	−15.3846	−21.4286	31.70732
2	Day trigger	10,190	14.5	1,478	77	1,138	44	650	23	340	84	1.91	11	163	11	163	9	2	6	18	9	10	90.8
2	Day OPU	9,920	16.2	1,607	81	1,302	50	804	25	402	87	2	11	177	11	177	8	1	5	14	6	7	95.19
2	%Δ	−2.64966	11.72414	8.728011	5.194805	14.41125	13.63636	23.69231	8.695652	18.23529	3.571429	4.712042	0	8.588957	0	8.588957	−11.1111	−50	−16.6667	−22.2222	−33.3333	−30	4.834802
3	Day trigger	6,540	19.9	1,301	88	1,145	41	534	46	599	90	0.89	6	78	6	78	17	1	13	21	17	17	86.9
3	Day OPU	6,360	18.6	1,183	89	1,053	44	521	42	497	90	1.05	6	71	7	83	16	1	14	20	15	16	87.5
3	%Δ	−2.75229	−6.53266	−9.06995	1.136364	−8.03493	7.317073	−2.43446	−8.69565	−17.0284	0	17.97753	0	−8.97436	16.66667	6.410256	−5.88235	0	7.692308	−4.7619	−11.7647	−5.88235	0.690449
Dual trigger group																							
1	Day trigger	8,610	23.6	2,032	85	1,827	66	1,341	18	366	89	3.67	7	142	7	142	2	4	2	7	3	3	25
1	Day OPU	8,480	23.1	1,959	84	1,645	61	1,195	20	392	88	3.05	9	176	9	176	2	1	4	6	4	5	47
1	%Δ	−1.50987	−2.11864	−3.59252	−1.17647	−9.96169	−7.57576	−10.8874	11.11111	7.103825	−1.1236	−16.8937	28.57143	23.94366	28.57143	23.94366	0	−75	100	−14.2857	33.33333	66.66667	88
2	Day trigger	6,580	29.3	1,928	77	1,485	52	1,003	22	424	84	2.36	9	174	9	174	2	2	10	12	9	10	99
2	Day OPU	7,870	27.7	2,180	82	1,788	52	1,134	25	545	89	2.08	6	131	6	131	5	1	15	16	17	17	99
2	%Δ	19.60486	−5.46075	13.07054	6.493506	20.40404	0	13.06082	13.63636	28.53774	5.952381	−11.8644	−33.3333	−24.7126	−33.3333	−24.7126	150	−50	50	33.33333	88.88889	70	0

OPU, ovum pick-up.

The % Δ in the CD4% (−3.8% vs. 9.0%) and CD4/CD8 (−14.3% vs. 11.0%) were lower in the Dual trigger group compared to the GnRHa group. Conversely, markers indicative of cytotoxic differentiation, such as CD3CD57% (75% increase vs. a 14% decrease), CD2CD57% (61.1% increase vs. 20.1% decrease) and overall CD57% (68.3% increase vs. −19.1%) were elevated post dual trigger compared to GnRHa group. Comparisons between GnRHa and dual trigger groups using the Mann–Whitney *U* test showed no statistically significant differences across immune parameters (all *p* = 0.20). Notably, CD57-related markers demonstrated complete directional separation (*U* = 0), being consistently higher in the dual trigger group Table 3). No OHSS cases were observed. However, conclusions should be restricted to hypothesis generation due to the lack of randomization and small sample size.

**Table 3:** The percentage difference (%Δ) between the value on Day-OPU (post trigger) and the values on Day-trigger (Day-OPU minus Day-trigger divided by Day-trigger*100).

	Immune profile				
	CD4%	CD4/CD8	CD3CD57%	CD2CD57%	CD57%
GnRH group					
Average	9.025295	11.06227	−14.1026	−20.1609	−19.1036
SD	4.037728	6.650769	20.63266	11.55037	12.22576
*p* value	0.2	0.2	0.2	0.2	0.2
*U* statistic	6	6	0	0	0
Dual trigger group					
Average	−3.78788	−14.3791	75	61.11111	68.33333
SD	5.35687	3.556271	35.35534	39.28371	2.357023

OPU, ovum pick-up; SD, standard deviation.

## Discussion

This small, exploratory proof-of-concept study explored the impact of GnRH agonist (GnRHa) vs. dual trigger (GnRHa plus hCG) on peripheral immune profiles in women undergoing oocyte cryopreservation. Results suggest that dual trigger is associated with a trend toward a cytotoxic immune shift, reflected by a lower CD4/CD8 ratio and increased proportions of highly differentiated, cytotoxic CD57+ T cell subsets. The overall adaptive immune and NK/B cell populations remained relatively stable. While these intriguing findings align with hCG's known effects on inflammatory mediators, they are purely hypothesis generating due to the study's inherent limitations.

Successful embryo implantation depends on a tightly regulated balance between pro-inflammatory and tolerogenic immune pathways that extends beyond the local endometrial environment to the systemic immune compartment. Circulating immune cells and cytokines dynamically traffic to the uterus, such that peripheral immune profiles can reflect and potentially influence endometrial receptivity. Prior research on ART immunity has focused primarily on systemic cytokines or endometrial leukocyte populations (14, 15); our approach extends such work to the peripheral lymphocyte compartment at the time of oocyte retrieval, before any confounding effects of embryo transfer or pregnancy. It is important to emphasize that immune adaptation during ART cycles is highly compartmentalized. Ovarian and especially uterine leukocyte phenotypes differ significantly from circulating populations (16, 17). Immune cell populations in these compartments, such as uterine natural killer cells, macrophages, and regulatory T cells, exhibit distinct phenotypes and functional roles not fully reflected in peripheral blood samples. For instance, while CD57 + subsets were elevated in peripheral lymphocytes, the significance of this increase for ovarian or uterine function is unknown. Peripheral blood findings provide only a partial perspective on immune events occurring within reproductive-specific microenvironments. Of notice, Alecsandru et al. (18) could not demonstrate any effect of OS on the uterine immune cell population or HLA-C binding in healthy women undergoing OS. Access to tissue-resident immune cells was not feasible in the current study, and therefore caution should be exercised in extrapolating these findings to ovarian or endometrial biology directly. Future studies are therefore needed to directly evaluate tissue resident immune cell populations during ART cycles, including follicular fluid leukocytes, granulosa or cumulus cells, and endometrial leukocytes. Such investigations may better elucidate the interplay between OS protocols, immune modulation, implantation mechanisms and OHSS risk.

The addition of hCG in dual trigger protocols has long been known to exert pleiotropic effects on the maternal immune system, contributing to both pro-inflammatory and tolerogenic changes required for implantation (19, 20). hCG exposure may stimulate cytotoxic NK cell activity, T effector responses, and specific subsets required for implantation and maternal fetal tolerance. However, excessive activation or imbalance in cytotoxic phenotypes may predispose to inflammatory complications, such as OHSS. The observed increase in cytotoxic markers (CD57+) with dual trigger may provide preliminary evidence of hCG's immune modulatory capacity, but it remains unclear whether these systemic changes are beneficial or detrimental to reproductive outcomes. Moreover, in cryopreservation cases, outcomes such as implantation rates and early pregnancy loss, which are highly dependent on immune adaptations, cannot be assessed directly due to the absence of embryo transfer.

GnRHa, on the other hand, induces a physiologic surge of both LH and FSH, but does not directly modulate immune responses at the maternal-fetal interface. The immune response following GnRH agonist trigger is less well characterized, but it does not appear to promote the same degree of local immune tolerance or regulatory T cell recruitment as hCG (21). Moreover, it appears to induce less systemic inflammation, as evidenced by lower levels of CRP and cytokines (2, 4).

The present study suggests that hCG administration promotes a shift towards a more cytotoxic and highly differentiated effector immune profile, potentially at the expense of, or in conjunction with, a rebalancing of adaptive helper functions, compared to solely GnRHa administration. There is an increase in cells directly capable of killing target cells (NK cells and highly differentiated cytotoxic T cells), accompanied by a relative shift in the balance of T helper vs. T cytotoxic cells. These could suggest an enhanced direct cell-mediated immunity.

hCG is a critical hormone for maternal immune tolerance of the semi-allogeneic fetus. While a cytotoxic profile might seem contradictory to tolerance, specific subsets of NK cells (e.g., uterine NK cells) are crucial for implantation and placental development. CD57 expression is commonly associated with highly differentiated cytotoxic effector cells, including subsets of both T cells and NK cells. Increased CD57 expression may indicate an enhanced capacity for direct cell-mediated cytotoxicity, which is consistent with observations of hCG's pro-inflammatory effects (19, 20). However, interpreting CD57 + cells as purely cytotoxic is an oversimplification. CD57 expression can also signify terminal differentiation, senescence, or exhaustion, particularly under conditions of chronic activation or immune homeostasis (22, 23). Some CD57 + subsets have been shown to exhibit regulatory functions, counterbalancing excessive inflammation (22). This complex heterogeneity in CD57 + phenotypes complicates their direct association with clinical outcomes such as implantation success or OHSS. Our discussion reflects a nuanced understanding of these immune subsets, avoiding assumptions unsupported by the peripheral data.

Furthermore, this shift towards a more cytotoxic/suppressive profile, while preventing rejection of the semi-allogeneic fetus, these overly cytotoxic response could also be detrimental, as it poses the known increased risk to develop OHSS (2, 4, 5). Conversely, the lower inflammatory response during GnRHa trigger may account for reduced OHSS incidence but potentially lower implantation rates due to insufficient immune activation.

### Strengths and limitations

This prospective, exploratory proof-of-concept, paired sample study design allows within individual assessment, reducing variability due to interpatient differences. The sample size was extremely small, with just 3 and 2 patients per group. Patient assignment to trigger strategy was based on clinical criteria, not randomization, introducing potential allocation and response bias. Furthermore, differing follicular response and hormone levels may independently affect immune parameters. For example, higher baseline hormonal levels or follicular activation may independently influence immune markers, and observed differences could reflect hormonal or ovarian factors rather than direct trigger effects. This confounding bias restricts the ability to isolate specific immunologic changes attributable to GnRHa vs. dual trigger strategies. Moreover, given this limited cohort and lack of randomization, the reported statistical outcomes using non-parametric tests must be interpreted cautiously. As such, suggested trends in immune modulation are exploratory rather than definitive, and comparisons between treatment groups require independent validation in larger, randomized studies. Furthermore, individual variability in baseline immune profiles highlights the need for more robust statistical power to confirm these observations.

No direct clinical outcomes (e.g., implantation, pregnancy) were assessed, as all cycles were oocyte cryopreservation. Additionally, only peripheral blood was sampled. Peripheral immune profiling cannot capture ovarian/endometrial microenvironments critical to implantation and OHSS risk. Extrapolating to reproductive outcomes therefore remains highly speculative. No cases of OHSS were observed in this small cohort. The findings should be therefore considered hypothesis generating and not generalizable to broader ART populations.

## Conclusion

Despite these limitations, findings from this study highlight potential immune differences that may be relevant for understanding ART outcomes and OHSS risk. This exploratory proof-of-concept pilot study suggests that the dual trigger protocol (GnRHa with hCG) is associated with a trend toward a cytotoxic shift in peripheral lymphocyte populations at the time of oocyte retrieval, compared to GnRHa alone. The observed cytotoxic immune shift with hCG might provide insights into its dual roles: stimulating implantation related immune activation but imposing a higher risk for inflammatory complications such as OHSS. Conversely, the immunologic profile elicited by GnRHa appears more stable, with fewer cytotoxic changes, which may explain this trigger's safety advantages but also its association with reduced implantation rates (5). Ultimately, these immune mediated effects create opportunities to tailor trigger strategies to individual patients, optimizing both clinical outcomes and health safety. While limited by a small sample size, these findings underscore the need for larger studies to fully elucidate the complex interplay between different trigger modes, specific immune cell subsets, and reproductive outcomes (24).

## Data Availability

The original contributions presented in the study are included in the article/Supplementary Material, further inquiries can be directed to the corresponding author.
